# Referee Acknowledgement for 2009

**DOI:** 10.1038/sj.bjc.6605730

**Published:** 2010-06-08

In this issue, we publish the names of all who reviewed manuscripts for us in 2009.

The Editor-in-Chief, Specialist Editors and everyone involved in publishing *BJC* would like to extend our sincere thanks to them for contributing their expertise and time. Our referees play an invaluable role ensuring that *BJC* continues to publish the high quality original papers and reviews that make it one of the world's leading oncology journals.

Abe, Yasuhito

Aboagye, Eric O

Abou-Alfa, GK

Abraham, Jame

Abrahm, Janet

Abramson, Jeremy S

Achen, Marc G

Acs, Geza

Adabag, AS

Adachi, Yasushi

Adams, Jo

Adams, Richard

Adolfsson, Jan

Aebi, Stefan

Agarwal, Roshan

Agelli, Maria

Aggarwal, BB

Ahern, Roger

Ahmed, Mansoor

Ajani, Jaffer

Akagi, Tadayuki

Akiba, Suminori

Al Moustafa, Ala-Eddin

Alaiya, Ayodele A

Alarid, Elaine

Al-Batran, Salah-Eddin

Albertsen, Peter

Albores-Saavedra, Jorge

Alexander, HR

Ali, Bettaieb

Alison, M

Allen, Naomi E

Al-Mukhtar, Ahmed A

Alsner, Jan

Alvarado, Carlos

Amant, Frederic

Amirkhosravi, Ali

Amstutz, Ursula

Anant, Shrikant

Andersen, MR

Andersen, Thomas T

Anderson, Alexander

Anderson, John

Ando, E

Andre, F

Andre, Thierry

Andreyev, H Jervoise N

Ansell, Stephen

Antillon, Minor

Apostoli, P

Arcaro, Alexandre

Arcasoy, Murat

Arkenau, Hendrik-Tobias

Armitage, James

Armstrong, Andrew

Armstrong, Jane

Armstrong, Michael

Armstrong, Miranda

Arribas, Joaquín

Arslan, Alan A

Ashcroft, Margaret

Ashworth, Alan

Assinder, SJ

Atanackovic, Djordje

Attard, Gerhardt

Auberger, Jutta

Augustine, Christi K

Ault, Kevin

Austoker, Joan

Autier, Philippe

Awada, Ahmad

Azuara, Daniel

Azzariti, Amalia

Bachinger, Hans Peter

Bafico, A

Bagley, Rebecca

Bagnato, Anna

Bajdik, Chris

Bajetta, Emilio

Baleja, JD

Balkwill, Fran

Ballman, Karla

Balter, Katarina A

Bamezai, RNK

Bandres, E

Banks, Lawrence

Baracos, Vickie E

Barbarroja, Nuria

Barbi, Maria

Barbolina, Maria

Bardeesy, Nabeel

Bardelli, Alberto

Barlati, Sergio

Barnes, Betsy

Barratt, J

Barritt, Greg

Baslund, B

Bast, Aalt

Batchelor, T

Bates, Richard

Bates, Susan E

Batra, Surinder K

Battaglia, Giorgio

Baudin, Eric

Baxevanis, Constantin N

Beaulieu, Jean-Francois

Beaver, Kinta

Begent, RHJ

Beier, CP

Bekaii-Saab, Tanios

Bellati, Filippo

Belli, Carmen

Bellone, Graziella

Bendall, Linda J

Benedict, Agnes

Bennett, Charles

Benson, Al

Bentrem, David

Berens, Michael

Berrier, Allison L

Berry, Donald

Bertagnolli, Monica

Bevan, Charlotte

Bevilacqua, Generoso

Bhakat, Kishor K

Bianchini, Giampaolo

Bicknell, Roy

Biege, Jaclyn A

Biel, MA

Biesma, B

Bifulco, Maurizio

Bigbee, William L

Bilchik, AJ

Bing, Chen

Biondi, Andrea

Birchmeier, Walter

Bird, N

Bíró, Tamás

Bisdas, S

Bishayee, A

Bishop, Tim

Bismar, Tarek A

Bissell, Mina

Black, Jennifer

Blana, Andreas

Blankenstein, Thomas

Blaydes, Jeremy

Bleyer, A

Blum, Hubert

Boccardo, Francesco

Bocci, Guido

Boddy, Alan V

Bodmer, Walter

Boeck, Stefan

Boerner, Julie

Bogen, B

Bokemeyer, Carsten

Bokstein, F

Boku, Narikazu

Bold, Richard

Boldrup, Linda

Bonavida, Benjamin

Bonecchi, Raffaella

Bonneterre, Jacques

Bonvalot, S

Bookman, Michael A

Borley, A

Bostrom, Peter

Bousquet, Corinne

Bouvet, Michael

Boven, Epie

Boyd, Douglas

Boyd, Norman F

Bozzetti, Federico

Brady, Joanne

Brambilla, Elisabeth

Bramhall, Simon R

Bramwell, Vivien

Brandes, Alba A

Brankovic-Magic, MV

Brauninger, Andreas

Bravo, Gina

Brentani, M

Brenton, James

Brewster, Abenaa

Bria, Emilio

Bridgewater, John

Brisken, Cathrin

Bristow, Robert G

Brodie, Angela M

Brodt, Pnina

Brody, Julia Green

Bronner, Christian

Brosens, JJ

Brown, Robert

Brunner, Thomas B

Brunton, Valerie

Bryant, Richard

Bujko, Krzysztof

Bukowski, Ronald

Buonaguro, Franco M

Burchill, Susan

Burger, Angelika

Burkhardt, Jeffrey H

Burum-Auensen, Espen

Burzykowski, Tomasz

Bushman, W

But, Igor

Butterfield, LH

Byers, Stephen W

Cairns, Ben

Cairns, Paul

Caldas, Carlos

Caldes, Trinidad

Callahan, Robert

Camidge, D Ross

Campbell, Ian

Campbell, Moray J

Canevari, Silvana

Cankovic, M

Cao, Brian

Cao, Jian

Cappuzzo, Federico

Caraglia, Michele

Carbone, Michele

Carling, Tobias

Carnero, Amancio

Carroll, Jason

Carroll, William L

Carson, William

Carver, Joseph

Cassidy, Jim

Castiglione, Guido

Catchpoole, Daniel R

Cavallo, Federica

Cawkwell, Lynn

Cebon, Jonathan

Celebi, JT

Cerchia, Laura

Cerutti, Andrea

Cervantes, Andres

Chabay, Paola

Chagpar, Anees

Chairatvit, Kongthawat

Chak, Amitabh

Chakravarti, Arnab

Chalmers, A

Chan, Francis KL

Chan, Stephen

Chang, Yu-Sun

Chanock, Stephen J

Charalabopoulos, Konstantinos A

Chatelut, Etienne

Chau, Ian

Chaudhuri, Gautam

Chen, Bin

Chen, Dong

Chen, Jeon-Hor

Chen, Li Tzong

Chen, RU

Chen, Xiaoxin

Cheng, Ann-Lii

Cheng, KW

Chhatwal, Jagpreet

Chi, Dennis S

Chieco, Pasquale

Chikumi, Hiroki

Chiosea, Simion

Chirieac, Lucian

Choi, Chel Hun

Choi, H

Choueiri, Toni K

Choyke, Peter L

Chris Lau, Yun-Fai

Christensen, JG

Chu, PG

Chu, Quincy

Chua, Daniel

Chung, YF

Church, James M

Chuthapisith, S

Clark, Matthew

Clarke, Robert

Clarke, Robert

Clarke, Stephen J

Cleton-Jansen, Anne-Marie

Cleveland, Rebecca J

Clifford, Gary M

Clifford, Steven C

Cocks, Kim

Coebergh, Jan-Willem

Cohen, Roger

Coiffier, Bertrand

Coleman, Robert

Coley, Helen M

Colleoni, Marco

Condorelli, Gerolama

Constine, Louis

Cook, Gary

Cook, Michael B

Cooke, Marcus

Cooper, Colin S

Cooper, Kumarasen

Cooperberg, Matthew R

Corless, CL

Correa, Candace

Correale, Pierpaolo

Corrie, Pippa G

Cortes, Javier

Costa, Luis

Costelli, Paola

Costello, Eithne

Courty, Yves

Coutant, Charles

Couvelard, Anne

Cox, Karen

Crabb, Simon

Cramer, Thorsten

Crawford, SM

Cristini, Vitorio

Cristofanilli, Massimo

Cross, SS

Croswell, Jennifer

Crowe, Francesca

Culig, Zoran

Culligan, Dominic

Culty, Martine

Cummings, Jeffrey

Cunningham, David

Cunningham, Moya S

Curley, Steven

Curtin, Nicola

Cusidó, Maite

Cuzick, Jack

Cybulski, C

Dahm, Philipp

Daidone, Maria Grazia

Daigo, Yataro

Dal Maso, Luigino

Dalbagni, G

D′Alessio, Giuseppe

Dalgleish, Angus G

Daly, Ann

Damjanov, I

Danesi, Romano

Danson, S

Darb-Esfahani, Silvia

Darby, Sarah

Dass, Crispin

Davies, Michael P

Davila, Monica

Davison, Joyce

Dayan, Frederic

De Angelis, Paula

De Azambuja, Evandro

De Bruïne, Adriaan P

De Giorgi, Ugo

De Greve, Jacques

De Groot, John F

De Hullu, JA

De Kerviler, Eric

De la Torre, Alejandro

De Lemos, ML

De Perrot, Marc

De Ridder, Mark

De Rinaldis, Emanuele

De Ruysscher, Dirk

De Vries, EGE

Dean, M

DeAngelis, Roberta

Debernardi, Silvana

Debucquoy, Annelies

Dedhar, Shoukat

Degani, Hadassa

Del Campo, Josep Maria

DeLeo, Albert B

Della Ragione, Fulvio

Dellapasqua, Silvia

DeMartin, Rainer

DeMeester, Stephen R

Denberg, Tom D

Denef, C

Deng, Chu-Xia

Denhardt, David T

Dent, AL

Dequanter, Didier

Derwinger, Kristoffer

Des Guetz, Gaetan

Desbois-Mouthon, Christèle

Desmedt, Christine

Dhingra, Ravi

Di Cosimo, Serena

Di Martino, Erica

Diamandis, Eleftherios P

Dias, Sérgio

Diederich, Marc

Dieli, F

Dietmaier, W

Dimopoulos, MA

Dincer, Y

Dirix, Luc Yves

Distel, Luitpold

Dittmer, Dirk P

Dixon, Simon

Dobrzanski, Mark J

Doehn, C

Dogliotti, Luigi

Dong, Zigang

Dopp, Elke

Dou, Q Ping

Dowdy, Sean C

Dowlati, Afshin

Downward, Julian

Dozois, Eric J

Drabkin, Harry

Drapkin, Ronny

Drappatz, J

Drexler, Hannes CA

D′Silva, Nisha

Du Bois, Andreas

Ducreux, Michel

Duff, Sarah E

Duffy, Michael J

Duffy, Stephen W

Duijm, Lucien

Dumontet, Charles

Dunning, Alison

Durrant, Lindy G

Dyer, Martin

Eberhardt, Wilfried

Eberhart, Charles

Eckel, Florian

Edelman, Martin

Edwards, Dylan R

Edwards, Joanne

Edwards, John G

Eeles, Ros

Eich, Hans Theodor

Eifel, Patricia

Eikje, NS

Eiser, Christine

Elenius, Klaus

Elias, Anthony

Elisei, R

El-Kareh, AW

Ellis, Ian

Ellis, Lee M

Ellis, Vincent

Ellisen, Leif W

Eng, Cathy

Engels, Eric

Engert, Andreas

English, Dallas

Eng-Wong, Jennifer

Esposito, Irene

Esteva, Francisco

Evans, D Gareth R

Evans, TR Jeffry

Fabi, Alessandra

Fadare, Oluwole

Faehling, Michael

Faingold, Dana

Fang, Bingliang

Faria, Mario Henrique

Farinati, Fabio

Favaudon, Vincent

Fayter, Debra

Fearon, Kenneth CH

Fehm, Tanja

Feliu, Jaime

Fenocchi, Eduardo

Ferlini, Cristiano

Fernandez de Mattos, Silvia

Fernandez, Conrad

Fernandez, E

Ferrandina, Gabriella

Ferrer, Montserrat

Ferrero, Maria Elena

Ferrucci, Leah M

Fiander, Alison

Fife, Kate

Figg, William

Figueroa, JD

Finke, James H

Finke, Jurgen

Fisher, Cyril

Flaherty, Keith

Flavell, David

Folprecht, Gunnar

Forootan, Shiva S

Fossa, Sophie D

Fossati, Roldano

Foulkes, William

Fountzilas, George

Franc, Brigitte

Franceschi, Silvia

Francken, Anne B

Frankel, Arthur

Frattini, Milo

Frazer, Ian

Fregnani, José

Frierson, Henry F

Friess, Helmut

Frizelle, Frank

Frommer, Marianne

Fruehauf, JP

Fujimoto, Toshio

Fukuda, Seiji

Furuse, Junji

Fusco, Alfredo

Gadducci, Angiolo

Gallagher, Michael

Gallego Melcon, S

Gallick, Gary E

Gallimore, Awen

Gallo, James M

Gallouzi, IE

Galon, J

Garbe, Claus

Gargett, Caroline

Gargiulo, G

Garzotto, Mark

Gasparini, Giampietro

Gatenby, Robert A

Gatter, Kevin C

Gatto, Barbara

Gautschi, Oliver

Gazdar, Adi

Gebbia, Vittorio

Gelderblom, Hans

Gennari, Alessandra

Gennari, L

Gennatas, Spyridon

Georgoulias, V

Gerke, V

Gerson, Stanton L

Gerss, Joachim

Gescher, Andreas J

Ghali, JK

Ghaneh, Paula

Ghiringhelli, Francois

Ghosh, Paramita

Giaccia, Amato

Giaccone, Giuseppe

Giantonio, Bruce

Gibbs, CP

Gibson, SB

Gielissen, Marieke

Gil, Jesus

Giles, Francis

Giordano, Antonio

Girotti, Albert

Glas, Annuska

Glaser, SL

Gleave, Martin E

Gleeson, Fergus V

Glimelius, Bengt

Glinsky, Gennadi

Gluck, Stefan

Gnant, Michael

Gnjatic, Sacha

Godfrey, TE

Goedert, James

Goetz, Matthew

Goh, Vicky

Goldenberg, David

Goldman, I David

Goldmann, Torsten

Goldstein, David

Gombos, Dan S

Goodall, Gregory

Goodell, V

Goodnough, LT

Gori, Stefania

Goydos, James

Gracey, J

Graff, Jeremy

Graziani, Grazia

Graziano, Francesco

Greco, Frank

Green, Jane

Greimel, Eva

Greten, Tim F

Groetzinger, Carsten

Gronberg, Henrik

Gross, Eva

Grossman, Ashley B

Grothey, Axel

Groves, Morris D

Gu, F

Guech-Ongey, M

Guedea, F

Guillonneau, Bertrand D

Guittet, Lydia

Guller, U

Gullick, William J

Guo, ZS

Gurney, Howard

Gurusamy, KS

Gustafson, Daniel

Gustafsson, Bjorn

Gusterson, Barry A

Gwyther, Steve

Hainsworth, John

Hall, Eric J

Halmos, Balazs

Hamilton, William

Hanash, Samir

Hanby, Andrew

Hancock, Barry W

Hannelius, Ulf

Happold, Caroline

Harada, Hiroshi

Harbour, J William

Hardwick, JC

Harenberg, J

Harmey, Judith H

Harrington, Kevin J

Harris, Eleanor

Hasan, Tayyaba

Hassett, Michael

Haun, Randy S

Hayashi, Kazuhiko

Hayes, Daniel F

Heidenreich, Axel

Hellstrom, Mats

Henschke, Claudia

Herlyn, Meenard

Herman, Eugene H

Hernandez, R

Hernando, Eva

Herold, Michael

Herold-Mende, Christel

Herrero-Martín, David

Herrington, C Simon

Herrlinger, Ulrich

Hess, Kenneth R

Hess, V

Hesson, Luke

Hezel, Aram

Hida, Toyoaki

Hirano, Takeshi

Hirsch, Fred

Hochaus, Andreas

Hochhauser, Daniel

Hoeben, RC

Hoekstra, HJ

Hoff, Geir

Hoff, Paulo

Hoffman, Richard M

Hofheinz, Ralf-Dieter

Hofstra, Robert MW

Hogendoorn, PCW

Hohenberger, Peter

Holdenreider, Stefan

Holen, Ingunn

Holmberg, LA

Holmberg, Lars

Homasson, Jean-Paul

Honma, N

Hooper, John D

Hopf, Carsten

Hopper, John L

Hori, Katsuyoshi

Hotta, Katsuyuki

Hotte, Sebastien

Hounsell, Alan

Houssami, Nehmat

Huang, Cheng-Long

Huang, Z

Huddart, Robert A

Hudis, Clifford

Hudson, Laurie

Huebner, Kay

Huet, G

Hughes, David

Hurwitz, Herbert

Husmann, DA

Hussein, Mahmoud

Hynes, Nancy

Hynes, Richard

Iacopetta, Barry

Ichikawa, Wataru

Ignatenko, Natalia

Ilic, Dragan

Imamichi, Yukiko

Inanami, O

Inoue, Akira

Inoue, Hiroshi

Irving, Julie

Isacke, C

Ishizuka, Mitsuru

Ito, Kiyoshi

Ittmann, Michael

Ivaska, Johanna

Iveson, TJ

Iyer, Renuka

Jablons, David

Jacobs, Elizabeth T

Jacobson, Arnold F

Jacot, William

Jain, Rakesh

Jakowlew, Sonia

Jänicke, Reiner

Jankoviæ, MM

Jankowski, Janusz

Jayadevappa, R

Jayson, Gordon C

Jelinek, Michael

Jenkins, Mark A

Jenkins, Valerie

Jensen, RL

Jerant, AF

Jeruss, Jacqueline S

Jess, Per

Jess, Tine

Jett, James

Jiang, Shi-Wen

Jin, Dong-Yan

Jirik, FR

Joab, Irene

Joel, Simon

Joensuu, Heikki

Joerger, Markus

Johnson, Bruce E

Johnson, Candace

Johnson, David

Johnston, Patrick

Jones, Arwyn T

Jones, Chris

Jones, Roger

Ju, Jingfang

Jung, Klaus

Jung, Manfred

Kabakov, Alexander E

Kacprowicz, RF

Kahan, Zsuzsanna

Kaina, Bernd

Kakimi, Kazuhiro

Kamen, Barton A

Kaneko, Michio

Kang, Mo K

Kang, SB

Kang, Won Ki

Kang, Yoon-Koo

Kannagi, Reiji

Karakiewicz, PI

Karanikas, V

Karapetis, Christos

Karpanen, Terhi

Karran, Peter

Kato, Hiroyuki

Kato, Kikuya

Katoh, Masaru

Kattan, Michael W

Kaufmann, Scott H

Kaye, Stanley B

Kayikcioglu, F

Keeley, Paul

Kellen, Eliane

Keller, ET

Kelly, Catherine Margaret

Kerbel, Robert S

Key, Timothy

Khan, Omar A

Khorana, AA

Kiaris, Hippokratis

Kiesslich, R

Kigawa, Junzo

Kim, Joseph

Kim, Mijung

Kinoshita, Yusuke

Kitawaki, Jo

Kitchener, Henry C

Kiura, Katsuyuki

Klampfer, L

Kleibl, Zdenek

Klymkowsky, MW

Knutson, Keith

Kobayashi, H

Kobayashi, Takashi

Kocher, Hemant M

Koelink, Pim J

Koenig, AM

Kohorn, Ernest

Koi, M

Kollmar, Otto

Konecny, Gottfried E

Kono, Koji

Konstantinopoulos, Panagiotis A

Kontovinis, LF

Koopman, Miriam

Korn, WM

Kornek, Gabriela V

Kotzerke, Jorg

Koutsilieris, Michael

Kovacic, Hervé

Krane, Louis Spencer

Kreicbergs, Ulrika

Krengli, Marco

Krishna, S

Krishnamurthi, Smitha

Krishnamurthy, Savitri

Kristensen, Gunnar B

Krop, Ian

Krug, Lee

Krzystek-Korpacka, M

Kucuk, C

Kuczyk, M

Kuerer, Henry

Kumar, Ashok

Kunz-Schughart, L

Kweekel, Dina

Kwong, Ava

Kwong, Joseph

Kyo, Satoru

Kyzas, Panayiotis

Lacey, James

Lage, Hermann

Lagos, Dimitrios

Lai, James CK

Lakhani, Sunil

Laking, George

Lambrechts, D

Lanvers-Kaminsky, C

Lappe, Joan

Larminat, Florence

Larsson, Susanna C

Lassmann, Silke

Latini, David

Law, Malcolm

Le, T

Lebeau, Jérôme

Ledermann, Jonathan

Lee, Chung

Lee, Ho-Young

Lee, Patrick WK

Lee, Se-Hoon

Lee, Siow Ming

Lee, Sug Hyung

Lee, Su-Jae

Lee, Ting-Yim

Lee, W Robert

Leenders, William

Leff, DR

Leffers, N

Lelong-Rebel, Isabelle

Leone, G

Leong, Stephen

Lesniak, Maciej

Leunen, Michele

Leung, Hing

Lev, Dina

Levi, Zohar

Levin, Myron

Li, Benjamin

Li, Daqing

Li, Lei M

Lianidou, ES

Lillard, James

Lin, Jin-Ching

Linch, David C

Lincz, Lisa F

Lind, Michael

Linder, Stig

Lindquist, SL

Lingwood, C

Liu, RS

Lively, Tracy

Loadman, Paul

Lofts, Fiona

Logan, Richard

Lokeshwar, Bal L

Lolkema, MPJK

Looijenga, Leendert

Lordick, Florian

Loupakis, Fotios

Lugli, Alessandro

Lukanova, Annekatrin

Lunt, Sarah Jane

Lurain, JR

Lurie, Galina

Lutgendorf, Susan

Luzzatto, Lucio

Lynch, HT

Ma, Ding

Mack, Jennifer

Madanat, Laura

Madani, Indira

Madhusudan, Srinivasan

Maehara, Yoshihiko

Mafune, Ken-ichi

Maggiolini, Marcello

Magne, Nicolas

Mahmud, Salaheddin

Mairs, Rob

Major, Eugene O

Maki, Robert

Makris, Andreas

Malagarie-Cazenave, Sophie

Malle, Ernst

Malouf, Gabriel

Maluf-Filho, Fauze

Mandusic, V

Mani, Sridhar

Manne, Upender

Manning, H Charles

Manson, M

Maranzano, Ernesto

Marbaix, Etienne

Marcickiewicz, Janusz

Marcus, Pamela M

Mareel, M

Marinaki, Anthony M

Markman, Maurie

Marlow, Laura

Marriot, Susan J

Marsh, Sharon

Martelli, Alberto

Martens, John

Martin, Francis L

Martin, J

Marty, Michel

Maskarinec, Gertraud

Mason, Bruce

Mason, Malcolm

Mason, Phillip J

Massarweh, Suleiman

Matei, Daniela

Mateo, Silvia

Matthay, Katherine

Mawdsley, Suzannah

May, F

Mayer, Thomas

Mayo, Juan

Mazda, Osam

Mazure, Nathalie

McAllister, Sean

McCulloch, Peter

McKeage, Mark

McManus, R

McMillan, Donald C

McNeish, Iain A

Mead, GM

Medema, Jean Paul

Meijer, Gerrit A

Memo, M

Meric-Bernstam, Funda

Messersmith, Wells A

Metcalfe, Chris

Meyer-Siegler, KL

Meyskens, Frank L

Middleton, Mark R

Midgley, Rachel

Mikami, Shuji

Miki, Yoshio

Mikula, Michal

Miles, David

Miletic, Hrvoje

Millar, Ewan KA

Miller, Robert C

Miller, Thomas

Milleron, Bernard

Milne, Roger Laughlin

Milner, JA

Min, YE

Minami, Yuko

Minamoto, Toshinari

Minchin, Rodney F

Minchinton, Andrew

Minn, Heiki

Minna, John

Mishra, Roshan

Missiaglia, Edoardo

Miyajima, Akira

Miyazono, Kohei

Modjtahedi, Helmout

Moehler, Markus

Molinari, Agnese

Mols, Floortje

Molven, Anders

Monden, Morito

Monk, Bradley

Montgomery, David Andrew

Moon, Chulso

Moon, Eun-Yi

Moon, Randall T

Moore, Cynthia

Moore, Patrick S

Moreno, Carlos S

Morente, MM

Morin, Pat

Morreau, Hans

Morrison, David S

Moss, Sue

Mottolese, Marcella

Mukhtar, Hasan

Müller, Volkmar

Munoz, Alberto

Murphy, Gillian

Murphy, Susan K

Murray, Graeme

Murray, Paul

Murray, Scott A

Nadji, M

Nahta, Rita

Naomoto, Yoshio

Narod, Steven A

Nath, Christa

Natsugoe, Shoji

Neckers, Len

Nelson, MA

Nelson, William

Nenutil, Rudolf

Neoptolemos, John

Neri, Dario

Nesland, Jahn

Neumann, Jens

Neuzil, Jiri

Newell-Price, J

Newton Bishop, Julia A

Newton, Robert

Nicoletti, MO

Nie, Daotai

Nieder, Carsten

Nijman, Hans W

Nirodi, Chaitanya

Nitti, Donato

Niv, Yaron

Njar, Vincent CO

Noh, Dong-Young

Noman, ASM

Numico, Gianmauro

Nur, Ula

Nyman, Jan

Nystrom, Lennarth

Obasaju, Coleman

Obermair, Andreas

O′Brien, Mary ER

Ochs-Balcom, Heather M

O′Connor, James PB

O′Connor, Rosemary

Oeffinger, Kevin

Ofner, CM

Oh, Sae-Ock

Oh, William

Ohkohchi, Nobuhiro

Ohnishi, Hirohide

Ohnishi, Takeo

Ohtsu, Atsushi

Okajima, Fumikazu

Okamoto, Isamu

Oliver, RTD

Olson, Michael F

Ong, CJ

Orloff, Mohammed

Orntoft, Torben F

Oter, Sukru

Ottensmeier, Christian

Ou, Y-H

Oue, Takaharu

Ouyang, Gaoliang

Oza, Amit

Pabst, Thomas

Page, Clive

Paggi, Marco

Pagliaro, Lance

Palmirotta, Raffaele

Palmqvist, Richard

Pals, Steven T

Pandolfi, Pier Paolo

Pantel, Klaus

Papagrigoriadis, Savvas

Paraskeva, Chris

Park, Keunchil

Park, Myung H

Park, Sook Ryun

Park, Y

Parkhurst, Maria R

Parkin, Donald Max

Parkinson, Ken

Parmentier, Marc

Parolaro, D

Paschke, R

Pascoe, Shane

Pasticier, Gilles

Paterson, Ian

Patnick, J

Paul, J

Pavlakis, K

Pavlidis, Nicholas

Pawelek, John

Pearson, Andrew DJ

Pecherstorfer, Martin

Peggs, Karl

Peltonen, Juha

Pelvris, John

Penel, Nicolas

Pennell, Nathan A

Pepper, Chris

Perini, Marcos

Perrone, Francesco

Perrotti, Michael

Peters, Godefridus

Peters, Ulrike

Peto, Julian

Peto, R

Petrioli, Roberto

Pfeiffer, Per

Pfreundschuh, Michael

Philip, Philip

Pickles, T

Pidgeon, Graham

Pien, Homer H

Piepoli, Ada

Pieraga, Jean Yves

Pierga, Jean-Yves

Pinkawa, Michael

Pintzas, Alexandros

Pinzani, Pamela

Pirker, Robert

Pisani, Paola

Plummer, Elizabeth

Podhajcer, Osvaldo L

Polesskaya, Anna

Pollak, Kathryn

Pollard, Steven M

Pollock, Pamela M

Polyzos, Aristides

Poon, Randy

Poon, Ronnie

Porter, RJ

Porter, WW

Portugal, José

Posner, JB

Pouyssegur, Jacques

Powell, Ned G

Powles, Thomas

Pozzo, Carmelo

Prestwich, Glenn D

Pritchard, Kathleen I

Procissi, Daniel

Protheroe, Andrew

Pucci, Sabina

Pugh, Christopher W

Puglisi, Fabio

Purmonen, Timo

Putzer, Brigitte M

Pyronnet, Stephane

Qin, Xinyu

Quaglia, Alberto

Queiroz, LM

Quelle, Dawn E

Rabbitts, Pamela H

Rack, Brigitte

Rafii, Shahin

Rai, Sajal

Rajasekaran, AK

Rakha, Emad

Ramon y Cajal, Santiago

Ramos, Maria

Rampling, Roy

Ranieri, Elena

Rao, Jianghong

Rao, Sheela

Rapidis, Alexander

Ratai, Eva-Maria

Ratcliffe, Peter

Ray, Swapan

Raymond, Eric

Real, FX

Reardon, David

Recio, Juan Ángel

Reddanna, P

Rees, Judy R

Rehli, Michael

Reich, Jerome M

Reichert, Janice M

Reinacher-Schick, Anke

Reis-Filho, JS

Ribas, Antoni

Ribatti, Domenico

Ricciardi, S

Rich, T

Richards-Kortum, Rebecca

Richardson, Des R

Rickles, Frederick R

Ridolfi, R

Ridsdale, Leone

Riess, Hanno

Rini, Brian

Rivoltini, Licia

Roach, Mack

Roberts, Dafydd L

Roberts, Fiona

Roberts, Mary F

Roberts, Sally

Roddam, Andrew W

Rodolfo, Monica

Romani, Massimo

Romero, Yvonne

Ronen, Sabrina

Rosen, Kirill V

Rosengren, Rhonda

Ross, Paul J

Rosser, Charles

Rosti, Gianantonio

Roth, Arnaud

Roth, Wilfried

Rouzier, Roman

Roxburgh, Campbell

Royce, Melanie

Ruano-Ravina, Alberto

Rubins, J

Rudat, Volker

Ruf, Wolfram

Rufini, V

Runnebaum, Ingo

RuPeng, Zhuo

Russell, Mason

Russell, Pamela J

Rutella, Sergio

Ruzzo, Annamaria

Ryan, Qin

Saba, Julie D

Sabatino, Susan

Sabharwal, Ami

Sadahiro, Sotaro

Safe, Stephen

Saijo, Nagahiro

Sakamoto, Junichi

Sakurai, Yoichi

Salazar, EP

Saldanha, G

Sallam, Maha

Saltz, Leonard

Samlowski, Wolfram

Sampson, John

Sandoval, Guillermo

Santin, Alessandro D

Santoro, Massimo

Sardanelli, Francesco

Sargent, Daniel

Sarid, Ronit

Sartorius, Carol

Sasako, Mitsuru

Satia, Jesse A

Sattler, Martin

Sauven, Paul

Savoldo, Barbara

Sawai, Hirozumi

Saylor, Philip

Scandurro, Aline B

Schafer, Beat

Schiff, D

Schimmer, AD

Schinkel, Alfred

Schittek, Birgit

Schlag, Peter

Schmalfeldt, Barbara

Schmid, Kurt Werner

Schmidt, Gerwin

Schmidt, Matthias

Schneider, CM

Schneider, Gunter

Schoemaker, Minouk J

Schoffski, Patrick

Schouten van der Velden, Arjan P

Schreiber, Robert

Schreiber, Stuart

Schröder, Wolfgang

Schroth, Werner

Schwartz, Lawrence

Schwarzer, Guido

Scott, Rodney J

Sebag-Montefiore, David

Segnan, Nereo

Sekeres, MA

Selbach, Matthias

Sellors, John W

Semba, Kentaro

Semiglazov, Vladimir

Sen, Subrata

Serrano-Olvera, Alberto

Seruga, Bostjan

Sessa, Cristiana

Setoyama, T

Severi, Gianluca

Seymour, Lesley

Shah, Manish

Shalowitz, David I

Shan, Baoen E

Shanker, Anil

Sharifi, Nima

Sheahan, K

Sherman, Mark E

Shih, Ya Chen Tina

Shimada, Hideaki

Shimo, T

Shipley, Janet

Short, Michael A

Shou, Chengchao

Siddik, Zahid H

Siegel, David

Sier, Cornelis FM

Sigurdson, AJ

Silasi, DA

Silver, Andrew

Simon, Richard

Simpson, Nicholas E

Simpson-Haidaris, Patricia

Singh, Gurmit

Sinha, R

Siu, Lillian L

Skirnisdottir, Ingiridur

Slaby, O

Sleeman, Jonathan

Sleijfer, Stefan

Slotman, Dr Ben

Smalley, Keiran SM

Smit, Egbert F

Sneddon, Sharon

Sneyd, Mary-Jane

Snozek, Christine

Soares, Paula

Sobrinho-Simoes, Manuel

Solassol, Jerome

Sommers, Benjamin

Sood, Anil

Soong, Richie

Sorahan, Tom

Soreide, Kjetil

Soto, José Luís

Span, Paul N

Speicher, DW

Speirs, Valerie

Spicer, James

Springer, Caroline J

Squires, Matthew

Sredni, B

Stadler, Zsofia

Stanley, FM

Stattin, Par

Stearns, Vered

Steel, C Michael

Stein, Ken

Stein, Kevin

Steinberg, Pablo

Stener-Victorin, Elisabet

Stenning, Sally

Stevens, Victoria

Stewart, Lesley

Stiller, Charles

Stocken, Deborah

Stoeber, Kai

Stoecklein, Nikolas

Stricker, Bruno H Ch

Strosberg, J

Stupp, Roger

Sturgis, Erich M

Stylli, Stanley

Sueltmann, Holger

Sun, Li-Chun

Sun, Shi-Yong

Sun, Yinghao

Sundin, Anders

Suprasert, P

Surbone, Antonella

Sutcliffe, S

Sutherland, Leslie C

Sutherland, Robert

Sverrisdottir, Asgerdur

Swain, Amanda

Swerdlow, Anthony J

Swerts, Katrien

Swinnen, Johannes

Szallasi, Zoltan

Tabi, Zsuzsanna

Tada, Hirohito

Tagawa, Masatoshi

Tahara, Eiichi

Takata, T

Taketani, Shigeru

Takeuchi, Hiroya

Takigawa, Nagio

Talieri, Maroulio

Tamimi, Rulla M

Tamkovich, Svetlana

Tanaka, Shinji

Tanji, Nozomu

Tanke, Hans

Tanyi, Janos

Taylor, Aliki

Taylor, Graham

Taylor, KL

Teare, Dawn

Telerman, Adam

Telesca, Donatello

Terada, Keith

Terpos, Evangelos

Tewari, Krish

Tham, Lai-San

Theodorsson, Elvar

Theriault, Richard

Theys, Jan

Thomas, David

Thompson, Timothy

Thorburn, Andrew

Tichelli, Andre

Tinelli, Raffaele

Tischkowitz, Marc

Tisdale, Michael J

Toffoli, Giuseppe

Tofilon, Philip

Toi, Masakazu

Tomilin, Nikolai V

Tornberg, Sven

Tornillo, L

Torti, SV

Tot, Tibor

Touboul, Emmanuel

Tozer, Gill

Travis, Ruth

Trédan, Olivier

Trivers, KF

Tsao, Ming-Sound

Tsatsoulis, A

Tselepis, Chris

Tsilidis, Kostas

Tsirlis, Theodore D

Tsuda, Hitoshi

Turley, Shannon J

Turner, Nicola J

Turturro, Francesco

Tward, Jonathan

Twelves, Chris

Tworoger, Shelley S

Tyldesley, Scott

Ueno, Hideki

Uldrick, Thomas

Unal, Bulent

Urcelay, E

Urieli-Shoval, Siimcha

Van Cutsem, Eric

Van Damme, Jo

Van de Velde, Cornelis JH

Van der Heide, Uulke A

Van der Steeg, Alida

Van der Zee, Ate

Van Deurzen, CH

Van Diest, Paul J

Van Endert, Peter

Van Kempen, Leon

Van Laarhoven, Hanneke WM

Van Leenders, GJ

Van Leeuwen, F

Van Tol-Geerdink, Julia

Vandekerckhove, Bart

Vapiwala, Neha

Varley, Claire

Varma, Hemant

Varticovski, Lyuba

Velikova, Galina

Verbeke, Caroline

Verma, Rama S

Verschraegen, Claire

Verslype, Chris

Viani, Gustavo

Victorson, David

Voest, Emile E

Von der Maase, Hans

Von Pawel, J

Von Wagner, Christian

Voortman, Jens

Vousden, Karen

Wactawski-Wende, Jean

Wagner, Lars

Wakabayashi, M

Wakeford, Richard

Walczak, CE

Wallgren, Arne

Walsh, Craig

Walsky, Robert L

Wang, Yan

Wang, Zhiwei

Ward, Tim H

Warnakulasuriya, Saman

Wasan, Harpreet

Watabe, K

Watine, Joseph

Watson, Alastair

Watson, Dennis

Watson, R William

Watson, Susan A

Watters, Dianne

Waugh, David JJ

Webb, Andrew

Weber, Frank

Weber, Klaus-Josef

Weekes, Colin

Wei, Jun

Weichert, Wilko

Weinshilboum, Richard M

Weisberg, Ellen

Welsh, Fenella

Wen, Patrick Y

Wendler, D

Wentzensen, Nicolas

Wesierska-Gadek, Jozefa

West, Robert B

Whang, Edward E

White, Shane C

Whiteside, Theresa L

Whitman, Gary

Wicha, Max S

Widmann, Christian

Wiemels, Joe

Wig, Jai Dev

Wilcock, Andrew

Wilking, Nils

Williams, Ann Caroline

Williams, Gareth Haydn

Wilson, Robin

Wilson, William

Wimberger, Pauline

Winter, Matthew

Wolf, Jurgen

Wolf, Katarina

Wong, Alice ST

Wong, Chris KC

Wong, Nathalie

Wong, Rebecca K

Wong, Sandra L

Wong, Yu-ning

Wood, Karen

Woodhall, Sarah

Woodward, Wendy A

Wright, Jim

Wulczyn, F Gregory

Wyer, Peter C

Wyllie, Andrew

Wymann, Matthias P

Xiong, Sidong

Xu, Haodong

Yamada, Tesshi

Yamamoto, F

Yanagisawa, Kiyoshi

Yao, Min

Yarnold, John

Yau, Thomas

Ychou, Marc

Ye, Bin

Yen, Yun

Yip, George W

Yoder, Mervin C

Yoh, Kiyotaka

Yokota, Jun

Young, M Rita I

Young, Robin

Yousef, George

Yuasa, Yasuhito

Zackrisson, Sophia

Zacksenhaus, Eldad

Zahra, Mark A

Zanetti, Roberto

Zangemeister-Wittke, Uwe

Zappa, Marco

Zeybek, Naciye Dilara

Zhang, Baolin

Zhang, Lin

Zhang, Wu

Zhao, KN

Zhou, Hongning

Zhu, Andrew X

Zielinski, Christoph C

Zimmerman, Michael A

Zlobec, Inti

Zoller, Margot

Zonderland, HM

